# Estimation of the duration between HIV seroconversion and HIV diagnosis in different population groups in French Guiana: Strategic information to reduce the proportion of undiagnosed infections

**DOI:** 10.1371/journal.pone.0199267

**Published:** 2018-06-22

**Authors:** Mathieu Nacher, Antoine Adenis, Florence Huber, Edouard Hallet, Philippe Abboud, Emilie Mosnier, Bastien Bideau, Christian Marty, Aude Lucarelli, Vanessa Morel, François Lacapère, Loïc Epelboin, Pierre Couppié

**Affiliations:** 1 Centre d’Investigation Clinique Antilles Guyane, INSERM 1424, Centre Hospitalier de Cayenne, Cayenne, French Guiana; 2 COREVIH Guyane (Coordination de la lutte contre le VIH), Centre Hospitalier de Cayenne, Cayenne, French Guiana; 3 Département des Maladies Infectieuses et Tropicales, Centre Hospitalier de Cayenne, Cayenne, French Guiana; 4 Département des centres délocalisés de prévention et de Soins, Centre Hospitalier de Cayenne, Cayenne, Cayenne, French Guiana; 5 Croix Rouge Française Centre Prévention Santé, Cayenne, French Guiana; 6 Hôpital de Jour Adultes, Centre Hospitalier de Cayenne, Cayenne, French Guiana; 7 Agence Régionale de la Santé de Guyane, Cayenne, French Guiana; 8 Service de Dermatologie Vénéréologie, Centre Hospitalier de Cayenne, Cayenne, French Guiana; National and Kapodistrian University of Athens, GREECE

## Abstract

**Background:**

Given the great efforts put into the strategic objective of reducing the proportion of HIV-infected patients that are undiagnosed, the aim of the present study was to review the temporal trends between 1997 and 2016 for median estimates of infection duration and median CD4 count at diagnosis for the main patient origins in French Guiana.

**Methods:**

CD4 cell count at HIV sero-conversion and square root of CD4 cell decline were obtained using the CD4 decline in a cohort of HIV-infected persons in the UK, fitting random effect (slope and intercept) multilevel linear regression models. Multivariate analysis used robust regression for modeling the delay between estimated HIV seroconversion and diagnosis and quantile regression for CD4 at HIV diagnosis.

**Results:**

The median interval between the estimated HIV seroconversion and HIV diagnosis was 8 years for patients fromBrazil, 4.5 years for those from Haiti, 6.6 years for those from Suriname, 3.3 years for patients from Guyana, and 3.1 years for French patients. A simple robust regression model with French patients as reference group adjusting for sex and age at the time of diagnosis showed that the interval was significantly longer for Brazilian (β = +3.7 years, P = 0.001), Surinamese (β = +4.2 years, P<0.0001), Haitian origins (β = +1.5 years, P = 0.049) but not for those originating from Guyana (β = -0.03 years, P = 0.9); Men independently had a longer interval than women (β = +3.5 years, P<0.0001).

**Conclusions:**

Despite great efforts in French Guiana regarding HIV testing both in terms of diversification and intensification we still need to tailor the offer to better reach the communities in need. These results should help authorities scale up and optimize initiatives to reduce the proportion of patients who are unaware of their infection. They also raise the question of the role of stigma and discrimination as a barrier to HIV testing in small communities, and further emphasize the importance of reducing it.

## Introduction

Since 1981 and the first description of AIDS, there has beendramatic progress in the development of antiretrovirals and treatment strategies. If all patients were effectively treated, the elimination of HIV would even be theoretically possible[1]. But in real life, undiagnosedHIV-infected patients contribute disproportionally to the epidemic. A major strategic goal is thus to continually reduce the proportion of undiagnosed HIV infections and to treat all diagnosed with HIV in order to reduce morbidity and transmission.

French Guiana is the French territory with the worst HIV epidemic[2]. Over 75% of patients are foreign born. The prevalence of HIV among pregnant women has been over 1% for 2 decades. AIDS incidence is ten times higher than the national average (21.6 per 100,000 *vs*. 2.1 per 100,000)[3,4]. The territory has access to all the newest antiretroviral drugs, PreP, free universal health care for all HIV patients and residence permits for foreign patients. The suspected drivers of the epidemic are sex work, crack cocaine use, and multiple concurrent sexual partnerships[2].

Males, older age groups and foreign born patients were diagnosed later on average than other groups[5]. In the past 5 years, HIV testing strategies have been diversified with ELISA lab testing, testing in emergency services, mobile testing centers, rapid testing at private practices, community based testing, and self testing[6]. The number of tests performed in French Guiana is also very high, over twice of what is performed in Mainland France (205 tests per 1000 inhabitants versus 81 tests per 1 000 inhabitants, respectively).[7]

Methods based on CD4 count decline have been used to estimate the probable country of infection and they have allowed to estimate the duration between seroconversion and HIV diagnosis.[8]We recently estimated that in French Guiana 44–64% of foreign born patients were infected within French Guiana.[9]

Given the great efforts put into the strategic objective of reducing the proportion of HIV-infected patients that are undiagnosed, the aim of the present study was to review the temporal trends for median estimates of infection duration and median CD4 count at diagnosis for the main patientorigins in French Guiana. The use of country of birth as a categorization category rests on the assumption that communities are often grouped and that community-based interventions would help in reducing the duration of the interval between seroconversion and HIV diagnosis and reducing the proportion of HIV patients unaware of their HIV infection thus slowing down transmission in these communities.

## Methods

### Study design

#### Setting and participants

Data on HIV patients has been available since 1989in French Guiana. Clinical, biological and epidemiological data are collected by specific trained research technicians in the DMI2 government software until 2008, and then in eNADIS/DATAIDS[10].

#### Inclusion criteria

We included patients who were followed in Cayenne, Saint Laurent and Kouroubetween 1997 and 2016 for whom the initial CD4 count at the time of diagnosis was available.

### Statistical methods

#### Time since seroconversion

The duration of infection was estimated for each patient. This estimation was based on the rate of CD4 decline, which depended on each person’s age and ethnicity, between the CD4 count at the time of diagnosis and the estimated CD4 count at the time of HIV infection. CD4 cell count at HIV sero-conversion and square root of CD4 cell decline were obtained using the CD4 decline in a cohort of HIV-infected persons, fitting random effect (slope and intercept) multilevel linear regression models, as originally described in the UK.[8,11]This allowed to calculate CD4 counts at the time of infection and the slope in an age and ethnicity-dependent variable.[8,11] Most migrants French Guiana are from African American ancestry thus the median CD4 count and interquantile range, and the slope of CD4 (square root) decline used were those calculated for black populations (median 487(IQR = 377–619)), and 0.2+0.02*age at diagnosis, respectively.[8,11] For persons from northern Brazil, who are very often of mixed Amerindian ethnicity median CD4 count and interquantile range were used, and the slope of CD4 (square root) for other ethnicities (median 538(IQR = 403–701)), 0.55+0.02*age at diagnosis (S1 Table).

The time between seroconversion and time at diagnosis was estimated using the formula [square root (CD4 at seroconversion)-square root(CD4 at HIV diagnosis)] / slope of CD4 decline.[8,11]

#### Statistical analysis

The statistical analysis was performed using Stata 13.0 (Stata Corp LP, College Station, TX, USA).

Descriptive statistics were computed. Mostly the estimation of time between seroconversion and HIV diagnosis was plotted per year of diagnosis to describe temporal trends. Median CD4 counts were also plotted to see if this objective measure was coherent with the estimated durations between infection and diagnosis. This was performed separately for patients originating from Brazil, France (French Guiana), Guyana, Haiti, and Suriname. The choice of these risk groups was based on the fact that most HIV-infected patients are of foreign origin and that in an operational perspective, identifying differences would help scale up HIV testing efforts. Comparisons used the ranksum test and the Kruskal Wallis test. Multivariate analysis used robust regression for modeling the delay between estimated HIV seroconversion and diagnosis and quantile regression for CD4 at HIV diagnosis.

#### Ethics and funding

Patients included in the FHDH give informed consent for using their case record data for research and publication of research results. Patient identity is encrypted before the data is sent to the Institut National de la RechercheMédicale (INSERM), which centralizes data from Regional Coordinations for the fight against HIV (COREVIH) throughout France. The cohort was has been approved by the Commission NationaleInformatiqueetLibertés (CNIL) since Nov 27th 1991 and has led to many international scientific publications. No specific funding was obtained for this study.

## Results

Overall, in 2016, there were 1383 patients actively followed in the database of whom 279 were of French origin, 607 were of Haitian origin, 419 were of Surinamese origin, 173were from Brazil and 173 from Guyana. The median interval between HIV seroconversion and HIV diagnosis was 3.5years (Lower-Higher estimates = 0.5–6.7) in women and 6.4years (IQR = 8–9.3) in men, P<0.001.

**Fig 1:**
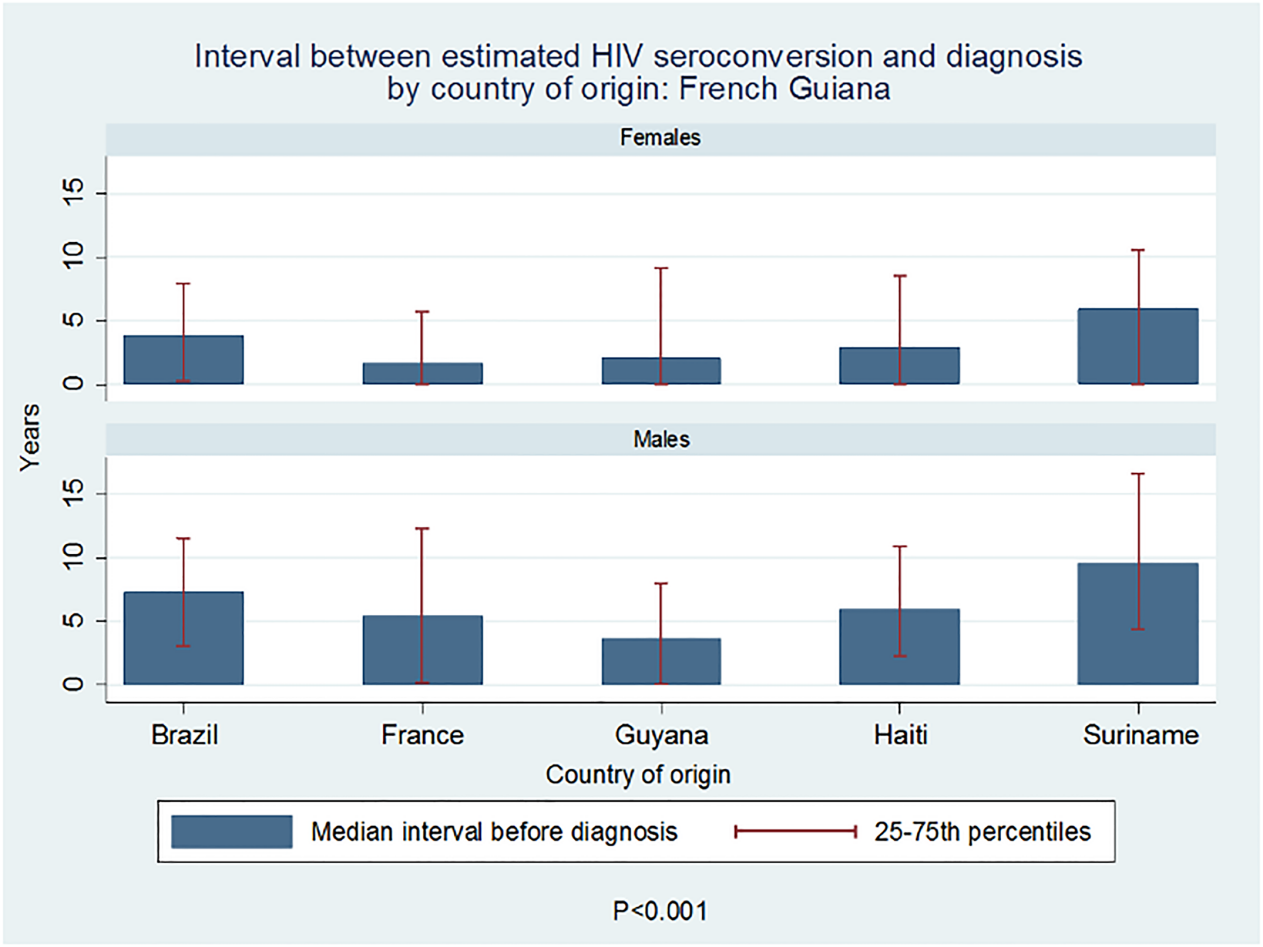
Median interval between the estimated HIV seroconversion and HIV diagnosis by country of origin.

The median interval between the estimated HIV seroconversion and HIV diagnosis was 5.1years for patients fromBrazil, 4.5 years for those from Haiti, 7.2 years for those from Suriname, 2.9 years for patients from Guyana, and 3.1 years forFrench patients (Fig 1).

These differences were statistically significant, P<0.0001 (Kruskal Wallis). A simple quantile regression model with French patients as reference group adjusting for sex and age at the time of diagnosisshowed that the interval was significantly longer Surinamese patients (β = +4.5years, P<0.0001), but not forthose originating from Brazil (β = +1.6 years, P = 0.16), Haiti(β = +1.3 years, P = 0.13) or Guyana (β = -0.5years, P = 0.6); Men independently had a longer interval than women (β = +3 years, P<0.0001). Some of the estimations were obviously distorted, leading to estimated dates of seroconversion that would have taken place after the actual diagnosis. Therefore, the median estimate seemed the more prudent option.

**Fig 2:**
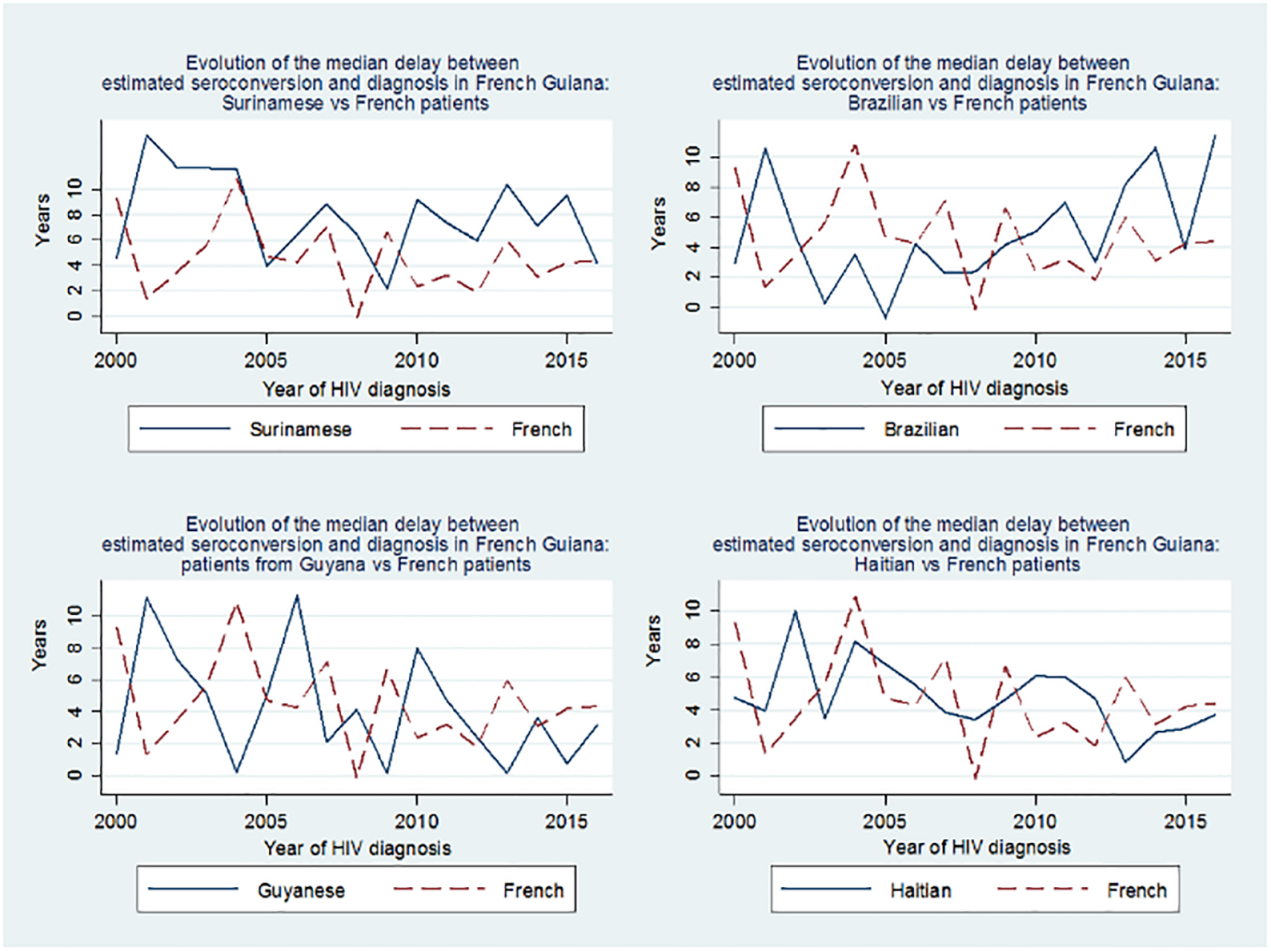
Temporal evolution of the median of the estimated delay between HIV seroconversion and diagnosis by country of origin.

Fig 2 shows the temporal evolution of the median of the estimated delay between HIV seroconversion and diagnosis for patients from Suriname, Brazil, Guyana, Haiti, relative to those from France (French Guiana). The data show that for Suriname and Brazil this delay was high, was stable for Suriname and seemed to increase for Brazil. For French citizens and for persons from Guyana the delay between seroconversion and diagnosis was shorter and seemed to decline. For patientsfrom Haiti, this delay was intermediate and seemed on a downward trend (Spearman’s Rho = -0.06, p = 0.2). These trends are also apparent when looking at the median CD4 count at the time of diagnosis with those from Brazil and Suriname having the lowest mean CD4 count at the time of diagnosis.

**Fig 3:**
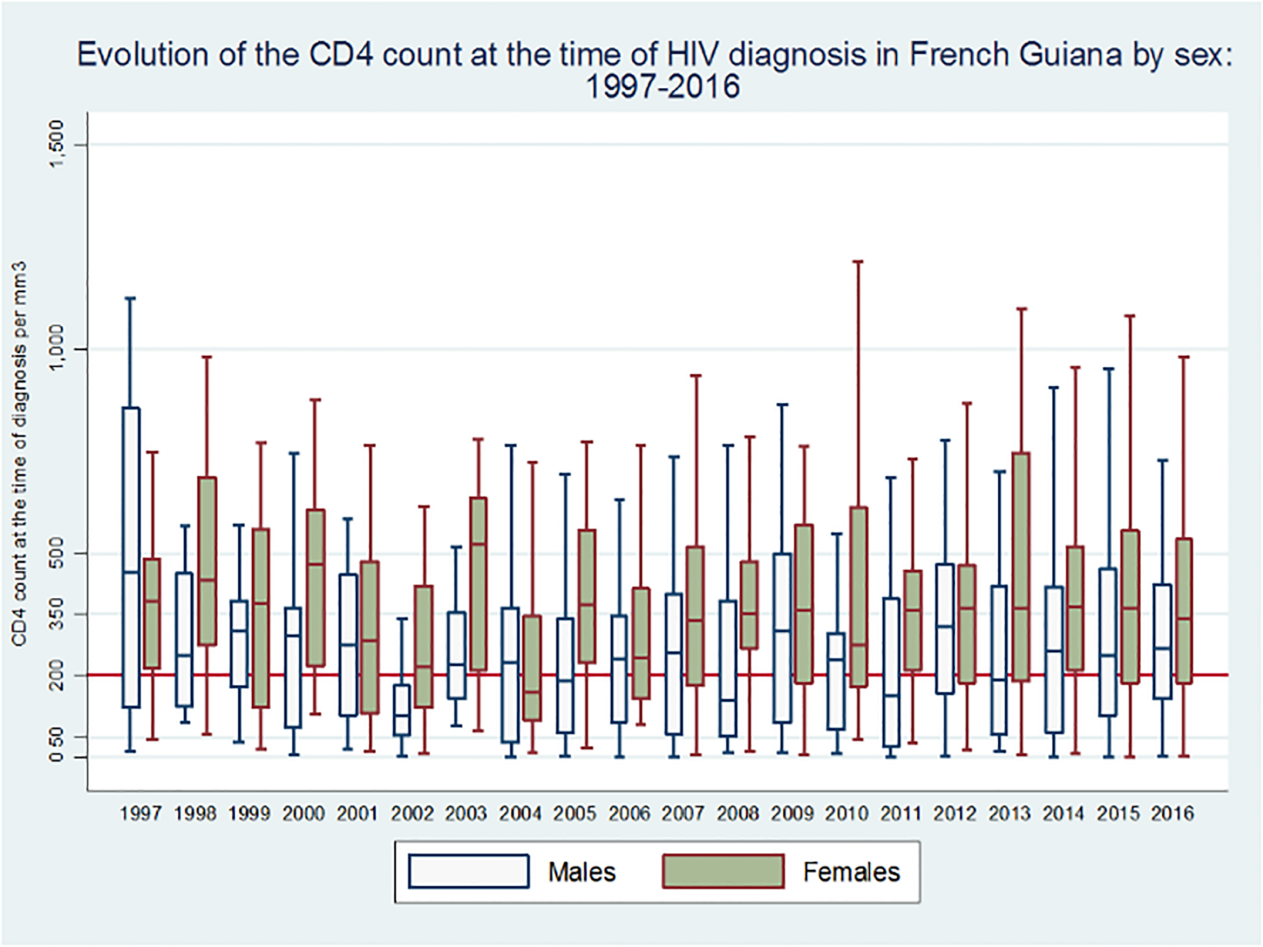
Temporal evolution of the CD4 count at diagnosis, by sex.

Fig 3 shows the CD4 count at diagnosis over time broken down by sex, which shows a regular difference with men testing later.

Quantile regression with CD4 count at diagnosis and country of origin with French patients as a reference group also found that patients from Suriname and Brazil had lower CD4 counts at the time of diagnosis. A quantile regression model with French patients as reference group adjusting for sex and age at the time of diagnosis showed that initial CD4 counts were significantly lower for patients from Brazil (β = -108 CD4 per mm^3^, P = 0.006) andSuriname (β = -136 CD4 per mm^3^, P<0.0001), but notfromHaiti(β = -39 CD4 per mm^3^, P = 0.16) orpatients from Guyana (β = +14 CD4 per mm^3^, P = 0.7).Fig 2 shows the temporal evolution of initial CD4 count at diagnosis among the main migrant groups and among French patients. There was a significant worsening of the situation for patients from Brazil (Spearman’s Rho = -0.21, p = 0.046), but not other countries of origin (France Spearman’s Rho = -0.08, p = 0.27; Haiti, Spearman’s Rho = -0.07, p = 0.17; Guyana, Spearman’s Rho = 0.02, p = 0.8; Suriname, Spearman’s Rho = -0.08, p = 0.18).

## Discussion

Undiagnosed HIV infections are a major driver ofthe HIV epidemic and thus reducing the interval between seroconversion and diagnosis through the improvement of HIV testing is a crucial strategic objective[1]. CD4 counts at the time of diagnosis gives crucial information about diagnostic delay. However, despite possible errors, transforming CD4 counts into a quantified estimation of the duration of the undiagnosed infection is not trivial because it frames the problem in a more meaningful way. The present results identify groups where HIV has a prolonged evolution before diagnosis. The results are also sobering because theyshow thatimprovement is at best slow. Furthermore, they suggest that among patients from Brazil, diagnosis is being performed later than before.

The fact that patients from neighboring Brazil and Suriname are tested so late, may reflect the fact that they are often persons living in remote areas in the interior, often patients living along the remote villages along the Surinamese side of the Maroni river, or more recently, Garimpeiros working in illegal goldmines.[12–14]They may be less informed about the benefits of HIV testing and perhaps less motivated than persons living in cities, and have fewer opportunities to get tested than urban populations. Stigma and discrimination in small communities may also be an important reason for not getting tested[15,16]. In these remote areas, scaling up of health education and testing efforts is recent and may still not be sufficient to impact the hidden infections’ reservoir. Patients from Guyana more often live in the Cities, originate from Georgetown, and have more opportunities and perhaps interest to get tested for HIV. Patients from Haiti had intermediate results, with after adjustments for sex and age, shorter intervals (1.3years later than French) than those from Suriname (4.5years later than the French) orfrom Brazil(1.6 years later than the French) but longer than patients from France (French Guiana) or Guyana. With median estimates between seroconversion and diagnosis of 5.1years among patients from Brazil, 7.2 years among patients from Suriname, 4.5years among patients from Haiti, 2.9 years among patients from Guyana, and 3.1years among French patients there is still a prolonged window of opportunity for HIV transmission. For patients of foreign origin, one hypothesis underlying the diagnostic delay would be that those starting to have symptoms related to their immunosuppression seek care in French Guiana because the French Health system is attractive to them. Patients from Guyana seem to go against this reasoning but this hypothesis could mainly concern those living in neighboring countries like Suriname or Brazil. However, a in a recent study in foreign patients from the Cayenne area it was estimated that 55% (Quartiles 45%–65%) of HIV-infected patients had acquired HIV in French Guiana (Manuscript in revision). This may go against the hypothesis of a special influx of the most immunosuppressed patients, and suggest that our interventions struggle to percolate through all population layers in French Guiana, especially the most in need.

The limitations of this study are that the estimation of the seroconversion date based on CD4 counts at diagnosis is an approximation. The calculations are based on ethnic groups, information that is not available in our data base and we made assumptions that may not always be right: all French patients are not of African descent but we used this hypothesis because creoles and maroons are the main population groups in French Guiana[17]. Although, the parameters in this method were calculated in the UK, populations from Latin America and the Caribbean were present and most of the viruses are also HIV1-B as in French Guiana. However, the slope parameters used and the estimations of median CD4 at diagnosis are susceptible to variation between ethnic groups and we cannot exclude distortions in our estimates.[18]Other authors have used methods relying on additional variables which may have been more precise[19–21], but because some of the information used was not available in our dataset, we used the above simpler method. Some of the computed infection dates were estimated to be after the diagnosis which is impossible. Thus calculated intervals should be taken with caution on an individual basis. However, at the population level, which was ourframe of reference, central measures may be an acceptable approximation. The above estimates are computed from HIV individuals who are diagnosed with the underlying assumption of proportionality of undiagnosed individuals. Possible limitationscould arise if the proportion of HIV infected persons who are diagnosed differed between groups.

UNAIDS 90x90x90 goal is within reach in French Guiana. Using the ECDC models, the estimated proportion of undiagnosed HIV-infections is *globally* estimated to be 15% in French Guiana, 91% of diagnosed patients in care are on antiretrovirals and 91% are virologically suppressed (Manuscript in review). The first part of the 90x90x90 objective still requires additional progress. There have been great efforts in French Guiana regarding HIV testing both in terms of diversification of the offer, and intensification of the numbers of tests performed. Each year, there are205 HIV tests per every 100 000 inhabitants, and increasing, versus 81 per 100 000 in mainland France.[22]We probably need to do even more in global quantity but also we need to tailor the offer to better reach the communities in need. We emphasize that looking at the country of origin is not aimed at stigmatizing foreigners, but at improving HIV diagnosis and care. These results should help authorities scale up and optimize initiatives to reduce the proportion of patients who are unaware of their diagnosis. They also raise the question of the role of stigma and discrimination as a barrier to HIV testing in small communities, and further emphasize the importance of reducing it[23]. Finally, all physicians should keep in mind that before HIV diagnosis, in most cases there are missed testing opportunities.[24]These long estimates of the undiagnosed period suggest that this is also very much the case in French Guiana, and that we should do even more.

In conclusion, the present analysis provides estimates for the duration between HIV infection and diagnosis in the attempt to gain additional insights to reduce late HIV diagnoses. Despite the potential limitations, the information is of public health interest. There have beenmany efforts to improve early diagnosis of HIV in French Guiana, but the results remain mostly humbling, with great differences between communities. The above results should incite the intensification of testing in the most vulnerable communities of French Guiana where the epidemic is presumably most active.

## Supporting information

S1 Table Parameters used to calculate the delay between HIV seroconversion and diagnosis for the main populations in French Guiana. (DOCX)

## References

[1] Sidibé M, Loures L, Samb B. The UNAIDS 90–90–90 target: a clear choice for ending AIDS and for sustainable health and development. Journal of the International AIDS Society. 2016 19(1): 21133 doi: 10.7448/IAS.19.1.21133 27424601 10.7448/IAS.19.1.21133 PMC4947868

[2] Nacher M, Vantilcke V, Parriault M, Van Melle A, Hanf M, Labadie G, et al What is driving the HIV epidemic in French Guiana? International journal of STD & AIDS. 2010 21: 359–361. 20498108 10.1258/ijsa.2010.009570

[3] Nacher M, Huber F, El Guedj M, Vaz T, Magnien C, Djossou F, et al Risk factors for death among patients in French Guiana: 1996–2005. HIV medicine. 2007 8: 472–474. doi: 10.1111/j.1468-1293.2007.00492.x 17760740 10.1111/j.1468-1293.2007.00492.x

[4] Cazein F, Lot F, Pillonel J, Le Strat Y, Sommen C, Brunet S, et al Découvertes de séropositivité VIH et de sida, France, 2003–2013. Bulletin Epidémiol Hebdomadaire. 2015 9: 152–161.

[5] Nacher M, El Guedj M, Vaz T, Nasser V, Randrianjohany A, Alvarez F, et al Risk factors for late HIV diagnosis in French Guiana. AIDS. 2005 19: 727–729. 15821399 10.1097/01.aids.0000166096.69811.b7

[6] Sangare I, Jolivet A, Adenis A, Adriouch L, Levy-loeb M, Dimanche S, et al Paid HIV rapid testing in general medicine private practice in French Guiana: a pilot project. Public Health.2017 151:23–26. doi: 10.1016/j.puhe.2017.06.002 28704721 10.1016/j.puhe.2017.06.002

[7] Cazein F, Le Strat Y, Sarr A, Ramus C, Bouche N, Pillonel J, et al Dépistage de l’infection par le VIH en France, 2003–2015. Bulletin Epidemiologique Hebdomadaire.2016 41–42: 745–8. http://invs.santepubliquefrance.fr/beh/2016/41-42/2016_41-42_2.html

[8] Rice BD, Elford J, Yin Z, Delpech VC. A new method to assign country of HIV infection among heterosexuals born abroad and diagnosed with HIV. AIDS. 2012 26: 1961–1966. doi: 10.1097/QAD.0b013e3283578b80 22781226 10.1097/QAD.0b013e3283578b80

[9] Nacher M, Adriouch L, Van Melle A, Parriault M-C, Adenis A, Couppié P. Country of infection among HIV-infected patients born abroad living in French Guiana. PLOS ONE.2018 13: e0192564 doi: 10.1371/journal.pone.0192564 29420591 10.1371/journal.pone.0192564 PMC5805311

[10] Pugliese P, Cuzin L, Cabié A, Poizot-Martin I, Allavena C, Duvivier C, et al A large French prospective cohort of HIV-nfected patients: the Nadis Cohort. HIV medicine.2009 10: 504–511. doi: 10.1111/j.1468-1293.2009.00719.x 19486189 10.1111/j.1468-1293.2009.00719.x

[11] Health Protection Agency Centre for Infections. Longitudinal analysis of the trajectories of CD4 cell counts. 2008. London: Health Protection Agency Centre for Infections. http://www.hpa.org.uk/web/HPAweb&Page&HPAwebAutoListName/Page/1201094588994

[12] Mosnier E Epidémiologie des maladies infectieuses et épidémiques en milieu isolé Amazonien.2017. Université de Guyane. https://tel.archives-ouvertes.fr/tel-01566849

[13] Mosnier E, Guiraud N, Epelboin L, Huber F, Adriouch L, Guarmit B, et al Diagnostic et prise en charge des PVVIH en zones isolées et frontalières en Guyane. Bulletin de Veille Sanitaire-Cire Antilles Guyane. 2015 11:10–16.

[14] Douine M, Mosnier E, Le Hingrat Q, Charpentier C, Corlin F, Hureau L, et al Illegal gold miners in French Guiana: a neglected population with poor health. BMC public health. 2017 18: 23 https://doi.org/10.1186/s12889-017-4557-4. 28716015 10.1186/s12889-017-4557-4 PMC5513330

[15] Van Melle A, Parriault M-C, Basurko C, Jolivet A, Flamand C, Pigeon P, et al Prevalence and predictive factors of stigmatizing attitudes towards people living with HIV in the remote villages on the Maroni River in French Guiana. AIDS care. 2015 27: 160–167. doi: 10.1080/09540121.2014.939607 25078678 10.1080/09540121.2014.939607

[16] Van Melle A, Parriault M-C, Basurko C, Jolivet A, Flamand C, Pigeon P, et al Knowledge, attitudes, behaviors, and practices differences regarding HIV in populations living along the Maroni river: particularities of operational interest for Amerindian and Maroon populations. AIDS care. 2015 27: 1112–1117. doi: 10.1080/09540121.2015.1032203 25909579 10.1080/09540121.2015.1032203

[17] Collomb G, Jolivet MJ. Histoires, identités et logiques ethniques: Amérindiens, créoles et noirs marrons en Guyane. 2008 Paris: CTHS, (18), 221 p. ISBN 978-2-7355-0662-0

[18] Pantazis N, Morrison C, Amornkul PN, Lewden C, Salata RA, Minga A, et al Differences in HIV natural history among African and non-African seroconverters in Europe and seroconverters in sub-Saharan Africa. PloS one.2012 7: e32369 doi: 10.1371/journal.pone.0032369 22412867 10.1371/journal.pone.0032369 PMC3295758

[19] Lodi S, Phillips A, Touloumi G, Geskus R, Meyer L, Thiébaut R, et al Time from human immunodeficiency virus seroconversion to reaching CD4+ cell count thresholds< 200,< 350, and< 500 cells/mm3: assessment of need following changes in treatment guidelines. Clinical Infectious Diseases. 2011 53: 817–825. doi: 10.1093/cid/cir494 21921225 10.1093/cid/cir494

[20] Pantazis N, Thomadakis C, del Amo J, Alvarez-del Arco D, Burns FM, Fakoya I, et al Determining the likely place of HIV acquisition for migrants in Europe combining subject-specific information and biomarkers data. Statistical methods in medical research. 2017 1:962280217746437. doi: 10.1177/0962280217746437 10.1177/0962280217746437 29233073

[21] Alvarez-del Arco D, Fakoya I, Thomadakis C, Pantazis N, Touloumi G, Gennotte AF, et al High levels of postmigration HIV acquisition within nine European countries. AIDS. 2017 31: 1979–1988. doi: 10.1097/QAD.0000000000001571 28857779 10.1097/QAD.0000000000001571

[22] Cazein F LS Y. Sarr A. Ramus C. Bouche N. Pillonel J. Lot F. Dépistage de l’infection par le VIH en France, 2003-2015// HIV testing in France, 2003–2015. Bulletin Epidémiologique Hebdomadaire. 2016 (41–42): 745–748.

[23] Yazdanpanah Y, Lange J, Gerstoft J, Cairns G. Earlier testing for HIV—how do we prevent late presentation? Antiviral therapy. 2010 15: 17 doi: 10.3851/IMP1526 20442457 10.3851/IMP1526

[24] Champenois K, Cousien A, Cuzin L, Le Vu S, Deuffic-Burban S, Lanoy E, et al Missed opportunities for HIV testing in newly-HIV-diagnosed patients, a cross sectional study. BMC infectious diseases. 2013 13:200 doi: 10.1186/1471-2334-13-200 23638870 10.1186/1471-2334-13-200 PMC3652743

