# Supplementary material for: Estimation of the duration between HIV seroconversion and HIV diagnosis in different population groups in French Guiana: Strategic information to reduce the proportion of undiagnosed infections
Source: PLoS One. 2018 Jun 22;13(6):e0199267. doi: 10.1371/journal.pone.0199267 (PMC6014655; doi:10.1371/journal.pone.0199267)
Supplement: S1 Table — (DOCX) [file pone.0199267.s001.docx]

Parameters used to calculate the delay between HIV seroconversion and diagnosis ([square root (CD4 at seroconversion)-square root(CD4 at HIV diagnosis)] / slope of CD4 decline) for the main populations in French Guiana.

| Origin | Median CD4 at seroconversion (IQR) | Slope of CD4 decline |
| --- | --- | --- |
| Brazil | 538(403-701) | 0.55+0.02*age |
| Guyana | 487(377-619) | 0.2+0.02*age |
| Suriname | 487(377-619) | 0.2+0.02*age |
| Haiti | 487(377-619) | 0.2+0.02*age |
| French Guiana | 487(377-619) | 0.2+0.02*age |
